# Toward a neural basis for peer-interaction: what makes peer-learning tick?

**DOI:** 10.3389/fpsyg.2015.00028

**Published:** 2015-02-10

**Authors:** Ian Clark, Guillaume Dumas

**Affiliations:** ^1^Nagoya University of Commerce and Business, Nagoya, Japan; ^2^Institut Pasteur, Human Genetics and Cognitive Functions Unit, Paris, France; ^3^CNRS UMR3571 Genes, Synapses and Cognition, Institut Pasteur, Paris, France; ^4^University Paris Diderot, Sorbonne Paris Cité, Human Genetics and Cognitive Functions, Paris, France

**Keywords:** socio-constructivism, peer-learning, motivation, intersubjectivity, reciprocity, cooperation, reward

## Abstract

Many of the instructional practices that have been advanced as intrinsically motivating are inherent in socio-constructivist learning environments. There is now emerging scientific evidence to explain why interactive learning environments promote the intrinsic motivation to learn. The “two-body” and “second person” approaches have begun to explore the “dark matter” of social neuroscience: the intra- and inter-individual brain dynamics during social interaction. Moreover, studies indicate that when young learners are given expanded opportunities to actively and equitably participate in collaborative learning activities they experienced feelings of well-being, contentment, or even excitement. Neuroscience starts demonstrating how this naturally rewarding aspect is strongly associated with the implication of the mesolimbic dopaminergic pathway during social interaction. The production of dopamine reinforces the desire to continue the interaction, and heightens feelings of anticipation for future peer-learning activities. Here we review how cooperative learning and problem-solving interactions can bring about the “intrinsic” motivation to learn. Overall, the reported theoretical arguments and neuroscientific results have clear implications for school and organization approaches and support social constructivist perspectives.

## INTRODUCTION

Recent conceptual developments and empirical research undertaken by interdisciplinary research groups (Redcay et al., 2010; Krill and Platek, 2012; Sakaiya et al., 2013; Schilbach et al., 2013) support the proposition that the reward-related networks in the human brain are recruited during cooperative social interaction. This is of particular salience to educators who seek classroom instruction and assessment methods that motivate their students to assist each other (Wood et al., 1976; Vygotsky, 1978), and sustain that assistance until the learning activity has been completed successfully. The relatively recent empirical work by social neuroscientists has helped to elucidate this matter through the use of a technique which records the brain activity of two (or more) individuals simultaneously (Montague et al., 2002; Babiloni et al., 2006). This technique, known as hyperscanning, has proved that brain activity is fundamentally different when we interact with others rather than merely observing them (Dumas et al., 2012a; Guionnet et al., 2012; Schilbach et al., 2006). This supports a common call for taking the role of social- and peer-interaction more seriously as an important driving force behind the construction of the individual motivation to learn in collective settings. Therefore, the central question which guides the construction of this paper is: what is the relationship between “live” social interaction and the reward-related networks of the human brain, which when activated reinforce the motivation to participate in product oriented peer-learning activities.

**FIGURE 1:**
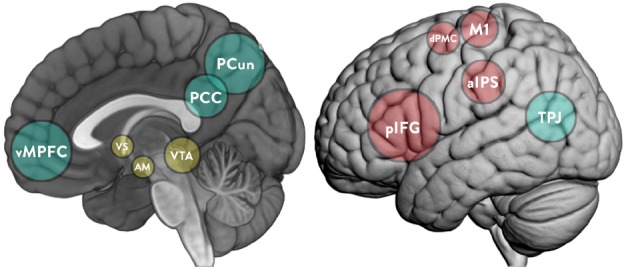
**Key brain structures implicated in the MNS (cyan), MENT (red), and mesolimbic reward system (yellow).** pIFG, posterior part of the inferior frontal gyrus; aIPS, rostral part of the inferior parietal cortex; dPMC, dorsal premotor cortex; M1, primary motor cortex; vMPFC, ventromedial part of the prefrontal cortex; PCC, posterior cingulate cortex; PCun, precuneus; TPJ, temporoparietal junction; VS, ventral striatum; VTA, ventral tegmental area; AM, amygdala.

“Second-person” (Schilbach et al., 2013) and “two-body” (Dumas, 2011; Nadel and Dumas, 2014) approaches argue that the social world and the world of the individual are interdependent, as seen in the foundational work of the influential developmental psychologist Vygotsky (1978, 1987), and that of more recent researchers (e.g., Rogoff, 2003). Neuroscience has also shown how motivation can emerge from the social world (Krach, 2010), and how social and reward-processing neural structures relate to each other (Ruff and Fehr, 2014). This article outlines the theoretical conception of an interactive approach to other minds and reviews evidence from neuroimaging, psycho-physiological, and psychological literature to rigorously explore the hypothesis that “intrinsic” motivation is a socially constructed phenomenon. This article reviews the literature on the social construction of motivation and the neuroscientific evidence that supports these perspectives. It discusses the functioning of two human brain networks commonly associated with social interaction, namely the mirror neuron system (MNS) and the “mentalizing” (MENT) system (see Figure 1). While the MNS is especially associated with goal understanding, empathy, and imitation, the MENT system is rather associated with higher-level cognitive processes such as understanding of other’s intentions and self-reference (Sperduti et al., 2014). Then, the article presents the concept of “agentic equity” and thematic discussions on the role of the mesolimbic reward pathway in cooperative learning, the developmental trajectory of the MENT and what this means for peer-learning. The discussions are focused around Bandura’s (2001) “agentic perspective”—a concept which explores the capacity to exercise control over the nature and quality of one’s life “within a broad framework of sociostructural influences.”

## THEORETICAL PERSPECTIVES ON THE SOCIAL CONSTRUCTION OF INTRINSIC MOTIVATION

Ryan and Deci (2000a) remark, “because intrinsic motivation results in high-quality learning and creativity, it is especially important to detail the factors and forces that engender versus undermine it.” When learners are intrinsically motivated they experience a sense of stimulation that compels them to persist with a learning task until its successful completion. Socio-cognitive studies on intrinsic motivation take an individualistic perspective, holding that “intrinsic motivation exists in the nexus between a person and a task” (Ryan and Deci, 2000a). Yet, as socio-constructivist theoreticans observe “they have also long recognized that contextual or social factors have a significant influence on these individual processes” (Walker, 2010). Accordingly, the influential socio-cognitive scholar Bandura (1977, p. 227) affirms that “cognitive development, of course is situated in sociocultural practices,” declaring such proclamations to be “no longer newsworthy.” Similarly, Ryan and Deci (2000b) emphasize the importance of interaction: “social environments can facilitate or forestall intrinsic motivation by supporting versus thwarting people’s innate psychological needs.” It is clear that socio-cognitive theorists no longer see the realization of instructional and motivational goals as an intra-psychological process unconnected to the social plane. Indeed, students’ react strongly to the social environment in schools, reporting emotions that range from apathy to anger (Gilman and Anderman, 2006). It can therefore be no surprise that students rarely report that they find studying to be intrinsically rewarding (Csikszentmihalyi and Larson, 1984).

Even though socio-cognitive theory recognizes the relevance of the social environment, motivation remains an individual phenomenon. More recently, theorists have suggested, after Vygotsky, that motivation is also social in nature (Walker, 2010). Sivan (1986) was the first educational theorist to elaborate on an individualistic theory of motivation. Her ideas received major impetus from Hickey’s (1997) article on contemporary socio-constructivist instructional perspectives. Hickey (1997) pointed out that “socio-constructivism is prominent in contemporary educational reform efforts” and urged for the expanded study of “new curricular approaches that follow from this perspective.” This has been supported by the recent empirical findings of social neuroscientists on the relationship between social interaction, neural reward (i.e., *dopamine* production: a hormone and neurotransmitter which plays a major role in reward-motivated behavior) and the motivation to learn (e.g., Redcay et al., 2010; Salamone and Correa, 2012; Sakaiya et al., 2013; Apps and Ramnani, 2014).

Socio-constructivist theoreticians contend that the motivation to learn is a socially constructed phenomenon (Hickey, 1997; Järvelä et al., 2010; Walker, 2010). Unlike their socio-cognitive counterparts, who try to understand the social through its residence in the mind of the individual, socio-constructivist theories give analytical and theoretical primacy to the social world over the individual world (Walker, 2010). In parallel, social neuroscientists argue for the “ontogenetic primacy of social interaction over observation” (Schilbach, 2014). For social constructionists there is a complex bi-directionality between individual learners and their social environment that may be described as a dynamic interdependence between the social and individual worlds. These worlds are distinguishable so that individual interactants maintain their specific identity, and do not merge into an “undifferentiated matrix” (Coelho and Figueiredo, 2003). Recent empirical findings from social neuroscience indicate that individual motivation and social inter-individual processes complement one another and tend to come into play concurrently (Adolphs, 2009).

From a social constructivist perspective “motivation is a socially negotiated process that results in an observable manifestation of interest and cognitive and affective engagement” (Sivan, 1986). In the specific context of the classroom many foundational studies have explored how social strategies of teachers (Brophy and Kher, 1984) and peers (Peterson et al., 1984) affect the motivation to learn. Thus, the role of social interaction would be emphasized in any discussion on motivation within a social constructivist perspective. Vygotsky’s zone of proximal development (ZPD), like Wood et al. (1976) concept of “scaffolding,” is a kind of assisted learning and “assisted learning is the method by which instructional and motivational goals are integrated” (Sivan, 1986). If the integration of these goals is to take place teachers, their students, and their peers need to develop intersubjectivity (Walker, 2010)—an interpersonal process that entails the making of subjective inferences in order to gain insights about the intentions and perspectives of others. In this Vygotskian perspective, one can never distinguish between an individual’s cognitive ability, the individual’s affective state, and the social environment (Rogoff, 1990).

There is compelling evidence that assisted learning holds considerable potential to improve student performance (Mynard and Almarzouqi, 2006; Kayi-Aydar, 2013), ensure outcome equity (Hedin, 1987; Benard, 1989; Reyes and Elias, 2011), enhance instructional efficacy (van Zundert et al., 2010) and, most significantly, motivate students to learn and think together more deeply (Sivan, 1986; Hickey, 1997; Mercer, 2000; Walker, 2010). There is therefore a deepening consensus on the proposition that cooperative “interactive styles” (Black and Wiliam, 2006) of classroom instruction are generally more effective than teacher-fronted didactic methods (Black and Wiliam, 1998; Assessment Reform Group [ARG], 1999; Organization for Economic Cooperation and Development/Centre for Educational Research and Innovation [OECD/CERI], 2005; Nicol and Macfarlane-Dick, 2006; Waxman et al., 2008; Cauley and McMillan, 2010; Clark, 2014).

## NEW PERSPECTIVES FROM THE INTERACTIVE TURN OF SOCIAL NEUROSCIENCE

Schilbach et al. (2013) note, “modern cognitive psychology has retained ‘methodological behaviorism’ from precisely the psychology it claims to have undermined” (also, Costall, 2006). This points at the enduring tradition of reductionism within social neuroscience, which separates the minds of the interactants from their observable behavior. Consequently, some recent neuroscientific studies (e.g., Gallotti and Frith, 2013) continue to separate neural activity (“social knowing”) from the social processes associated with that activity. Such studies focus on the use of, what Becchio et al. (2010) call, “isolation paradigms” in which participants are required to merely observe others, or think about their mental states as a detached observer. This approach highlights the shortcomings of a reductionist approach contend “second person” (Timmermans et al., 2013) and “two-body” (Dumas, 2011; Nadel and Dumas, 2014) neuroscientists. Recent empirical evidence (e.g., Cleeremans, 2011; Timmermans et al., 2012) even support that meta-cognitive skills, such as reflection, are acquired *during* social interaction. Our brain’s biological functions are thus constantly molded by internal reflection and external feedback as we attempt to model (or simulate) other minds during an interaction.

Passive/observational learning entails more limited neural activity than social-interaction (Schilbach et al., 2013). In a socio-constructivist “two-body” or “second-person” perspective interactive learning requires an intricate negotiation between people which recruits the neural processes underlying reciprocal social interaction, and involves both affective and cognitive aspects (Sakaiya et al., 2013). It is through this interaction that students learn to be conscious of their own actions. The resultant meta-cognitive reflection helps learners to exercise control over their environment as they attempt to realize their personal and learning goals (Bandura, 1997; Clark, 2012). The development of a meta-cognitive system not only guides the establishment of new behavioral routines, but also help monitoring their quality and progress over time (Chein and Schneider, 2012).

Rewarding social interactions between an individual and the social environment in which they are “embedded” is central to the overall purpose of this review (and will be revisited in a later section on dopaminergic reward). Early studies (Farroni et al., 2002) found that a preference for social engagement emerges as early as 2–5 days after birth. Even at this age, it is emotionally rewarding for infants to look at faces with eyes looking directly at them. Schilbach et al. (2013) note that when individuals are not emotionally engaged they cannot be expected to gain intersubjectivity—a necessary condition for rewarding peer-learning to occur. Intersubjectivity is the extent to which the “hidden” ideas, intentions and values of one participant are accessible to, understood and reciprocated by the other. A stable inter-subjective state is typically unavailable, so this is a continuous process of observation and inference at the intrapersonal level, and of social negotiation at the interpersonal (Fuchs and de Jaegher, 2010). This continuous process creates the conditions necessary for the integration of motivational and instructional goals (Sivan, 1986), or the “collaborative ZPD […] best understood as involving mutual adjustment and appropriation of ideas” (Goos et al., 2002; cf. Vygotsky, 1978, 1987).

Damon’s (1984) declaration that peer-learning is a “robust phenomena” was made many years prior to the modern neuroimaging procedures available today which have empirically demonstrated why assisted learning motivates people to work and learn together. Yet, even 30 years on, Baines et al. (2007) suggest that peer-learning remains a neglected area by teachers who typically plan for their interactions with students, but not for interactions between and among students. Tiknaz and Sutton (2006) found that peer discussions took place only once or twice in the school year. This was due to time constraints (often cited as a reason; see Organization for Economic Cooperation and Development/Centre for Educational Research and Innovation [OECD/CERI], 2005), complex assessment language, and mistrust of students as competent assessors. These teachers are missing the opportunity to motivate their students through expanded opportunities for participation. Social neuroscience suggests a close functional interaction between the social and emotional/motivational systems in the human brain (Lieberman, 2007; Sakaiya et al., 2013). This offers an opportunity for educators to reflect on classroom strategies which capitalize on learners’ self-reported preference for cooperative learning activities (Assessment Action Group/AiFL Programme Management Group [AAG/APMG], 2002–2008; Willis, 2010).

## THE POSITIVE EMOTIONAL VALENCE OF COOPERATIVE INTERACTION

The origin of the fundamental human need to belong has been suggested to originate in the advantage of cooperative over individualistic work performance (Baumeister and Leary, 1995; Wagner et al., 2014). The preference for cooperation supports a theory of “emotional-motor resonance” (Preston and de Waal, 2002) that neuroscientists propose as a “phylogenetically early system for empathy” (Molnar-Szakacs and Uddin, 2013). The term “resonance” implies a cognitive tension “between” learners as they seek mutual insights, jointly monitor the interaction and adapt to each other’s needs as learners. This shared, and ideally equitable tension, is represented as brains activating in the same areas as they interact (Jackson et al., 2006; Dumas et al., 2012a). Indeed, the intrinsic motivation to cooperate is so pronounced that “when [peers] share visual information in an interpersonal situation, they immediately coordinate their movements even when instructed to be intentionally uncoordinated” (Issartel et al., 2007). Accordingly, Sakaiya et al. (2013) found that: (a) the intensity of emotion associated with reciprocal peer-interaction presents in the mesolimbic dopamine reward system of the brain; and, (b) intersubjectivity is essential. Indeed, without intersubjectivity cooperative human relationships appear impossible (Sakaiya et al., 2013; Schilbach et al., 2013). The quality of intersubjectivity is determined by the predictability and stability of the interaction (Allen and Williams, 2011). Where unpredictability exists the motivation required for effective peer-learning diminishes, and the collaborative ZPD remains inaccessible because learners cannot assist each other until they have mutual insights into each others’ intentions and motives.

Learners experience positive feelings in anticipation of mutual interaction (Salamone and Correa, 2012), and of course, during an interaction as learners feel motivated to create and capitalize on opportunities to collaborate together in order to solve a particular problem (Redcay et al., 2010). Moreover, since the reward system also reacts to the value of reward received by others (Apps and Ramnani, 2014), learners can mutually detect and experience others’ reward during social interactions thus creating an additional mutual fulfillment during peer-learning. These changes in brain-chemistry are particularly important in motivating young learners to delve more deeply as they think and learn together.

Socio-constructivist theories posit that motivated learners are participants in interactive (Black and Wiliam, 2006; Schilbach, 2014) or cooperative relationships (Storch, 2002; Schilbach et al., 2013). Storch (2002) asserts that a collaborative relationship means much more than two or more learners working together. From this perspective cooperation is measured along two dimensions: equality and mutuality. Equality refers to the level of authority or *control* over the interaction. If learners are to assist each other then they need to demonstrate the ability to take direction from each other equally by a process of interactive turn-taking. Mutuality means the extent of *engagement* between each other’s contributions so that peers who exhibit a high level of mutuality assist each other by sharing ideas and giving feedback. From the realm of social neuroscience, Schilbach et al. (2013) present a similar theoretical model for rewarding and successful peer-assistance. Schilbach employs “emotional engagement” on the horizontal axis and “social interaction” on the vertical axis in parallel to Storch’s (2002) preference for “equality” and “mutuality.” Storch’s (2002) contention that effective thinking and learning is not a matter of people simply working together is consonant with that of Schilbach et al. (2013), who found that “intricate reciprocal reactions” emerge. Reciprocity motivates learners by recruiting the reward circuitry in the human brain, which encourages, sustains, and deepens individual peer-learning. This has been empirically demonstrated by Krill and Platek (2012) during a task requiring the participants to interact cooperatively in order to learn how to negotiate their way through a maze. In support of the long-standing findings of social learning theorists (e.g., Johnson and Johnson, 1996), they concluded that such learning activities “may be more rewarding under conditions of real time cooperation.”

The design and implementation of cooperative learning activities is empirically supported by the interactive, constructive, active, passive (ICAP) theoretical model that differentiates student engagement in learning tasks by categorizing students’ learning strategies as Interactive, Constructive, Active, or Passive (Chi, 2009; Menekse et al., 2013). It is founded on theoretical assumptions about how those strategies relate to different cognitive processes. The ICAP hypothesizes that Interactive activities will produce better learning outcomes than Constructive activities, and that all more effective than Passive learning strategies so that I > C > A > P. Interactive (I) engagement entails learning together and is, although not explicitly stated in Chi’s work, entirely consonant with social constructivist theory. A Constructive (C) strategy may be self-explaining, or creating a concept map in order to generate new knowledge. Active (A) behaviors include highlighting a textbook chapter and correspond to the internalization of new knowledge. Observational strategies would be considered Passive (P), corresponding to the process of storing knowledge. There is empirical support for the ICAP hypothesis, although the Interactive category carries a caveat (Menekse et al., 2013). That is that engagement should only be considered Interactive, and therefore rewarding, when both individuals in an interaction are being cooperative.

## NEURAL GROUNDING OF SOCIAL COGNITION

The next sections will explore two important neural systems implicated during social- and peer-interaction (see Figure 1). These are, (a) the “MNS”; and, (b) the “MENT system.” Research on the latter began as early as 1978 with the seminal work of Premack and Woodruff (1978). The understanding of the MNS/MENT relationship, the self, and internal/external stimuli advanced recently (Qin and Northoff, 2011; Sperduti et al., 2014) but require further methodological and theoretical developments (Dumas et al., 2014; Schilbach, 2014). While these systems are connected, they seem to operate at different levels of neural complexity. The MNS regulates low-level simulation processes, or *externally focused processes* related to one’s own or others’ visible expressions, actions and emotions. The MENT is involved in higher-level inference-based “MENT” processes, or *internally oriented processes*, which build a mental-model of others’ inner affective and cognitive, states; more clearly understood as an evaluation or reflection function (Uddin et al., 2007). Of course, this separation between MNS and MENT is also linked to a disparity of conceptual and methodological choices in social neuroscience (Schilbach, 2014). Both MNS and MENT are bidirectionally coupled and a full account of social cognition requires integrating both systems in different social context and across development (Dumas et al., 2014).

The MNS and MENT integrate internal and social information to achieve self- and other-understanding (Molnar-Szakacs and Uddin, 2013; Marchetti and Koster, 2014; Sperduti et al., 2014). The coordinated activity of these systems, therefore, provides the neural basis for more effective learning interactions that lead to improvements in meta-cognitive functioning (i.e., reflection). Such improvements are the consequence of the spontaneity inherent to authentic learning interactions which motivate people by activating the reward-related networks (Guionnet et al., 2012) and modifies substrates through neuro-plasticity or molding (Allen and Williams, 2011). Such inter-individual neural molding may explain over time the reinforcing of both anatomical and functional similarity between human brains and thus facilitating our propensity to interact socially with others (Dumas et al., 2012b).

Since MNS and MENT work together to provide a neural basis for social cognition (Sperduti et al., 2014), they pattern our capacity to interact with others and attain personal and learning goals in often challenging social situations (e.g., schools). Recent developments in social neuroscience confirm that mutual social empathy and engagement is a key pre-requisite (e.g., Schilbach et al., 2013) for the kinds of learning interactions that “waken a whole series of functions that are in a stage of maturation lying in the zone of proximal development” (Vygotsky, 1987, p. 212). The MNS and MENT and how they motivate individuals to learn in collective settings is the focus of the subsequent section of this article, beginning with the lower-level simulation processes executed by the MNS.

## LOWER-ORDER SOCIAL PROCESSING: THE “MIRROR NEURON SYSTEM”

The MNS (see Figure 1) is a neural network of “mirror neurons” which responds when we perform an action, and when we see that action being performed by others. The mirror neurons thus tend to be more active during cooperative interactions (Newman-Norlund et al., 2007). During social interaction, the MNS mechanisms unify the sensory perception of an action or emotion and the execution of a (re)action (Sandrone, 2013). More specifically, the MNS detects and monitors the spontaneous and incongruent “affordances” (momentary cues inviting immediate feedback) required for effective peer-interaction to take place (Schilbach et al., 2013).

While the MNS is fundamental for the study of the self in relation to others, and therefore fundamental to social cognition, it is considered a lower-order neural network due to its superficial interpretive function (Sandrone, 2013). The MNS is a conceptually behavioral system in that a literal interpretation would mean that we simply mirror the actions of others (those Newtonian relationships of which reductionists dream). This kind of predictability is evident in machines and simple life forms, but not among humans who live a complex social life (White, 1984; Clark, 2012). The MNS is important in comprehending the intentions of others by processing the sensorimotor or observable actions and emotions of others. It therefore helps people to recognize others as intentional beings; in this case learning-partners (Marchetti and Koster, 2014), and provides the basic platform from which dialogic peer-learning interactions may emerge. Even for young learners of limited social experience the “mirror-like processes” are strongly influenced by complex self-perspectives and experiences, and also by how they perceive the intentions of others (Meltzoff, 2005; Gazzola et al., 2007; Molnar-Szakacs and Uddin, 2013). These findings suggest that the MNS begins construction of the social foundation required for peer-learning (see Cook et al., 2014). It is becoming clear that this construction is a joint process; a fundamentally social process, and a crucial one that sustains cooperative verbal interaction.

The development of social “affordances” (Schilbach et al., 2013) into genuine opportunities for learning is dependent on the meta-cognitive awareness of students’ and the self-belief that their efforts will result in success (self-efficacy). For each momentary action or emotion we observe, the MNS models or simulates these states internally before preparing and executing a reaction. The MNS is by no means an exclusively visual system, as it responds to other environmental stimulation, e.g., sounds from which humans obtain information about other persons’ feelings and intentions (Caetano et al., 2007). As such, when dialog associated with someone else’s actions is listened to, the MNS is recruited (Molnar-Szakacs and Uddin, 2013), creating a “neural resonance” during an interaction (Dumas et al., 2010; Marchetti and Koster, 2014). Cooper et al. (2012), for instance, observed that when others are observed receiving a reward the reward centers of the observers’ brains are recruited as well (but to a lesser extent of course). Similar phenomena can be observed with empathy: when we observe that others are in physical or emotional pain the observers’ brains react as it was experiencing pain as well, running a sort of background simulation (Decety and Lamm, 2009).

The MNS is fundamental to the early development of intersubjectivity. Heyes (2010) and Schilbach et al. (2013) found that the neuro-plasticity of the MNS can be transformed through frequent exposure to the sensorimotor inputs of others. This means that, “the MNS, even in adulthood, can be reconfigured through sensorimotor learning” (Schilbach et al., 2013). So, when learners are mutually engaged on a frequent basis, the simulation-routines of the MNS may improve so they become a more stable basis for the preparation and execution of social and learning interactions. The fluid developmental trajectory of the MNS supports the widespread implementation of “relational skills training” (Blatchford et al., 2006), or other socio-emotional strategies (Collaborative for Academic, Social, Emotional Learning [CASEL], 2006) in public schools because they have the potential to support students’ identities as successful learners. The “early empathy” made possible by MNS processes is fundamental to the peer-learning relationships which provide the bedrock on which higher-order thinking and learning takes place (Clark, 2012).

## HIGHER-ORDER SOCIAL PROCESSING: THE “MENTALIZING” NETWORK

The MENT of self and others are closely “related processes that are crucial to navigating the social world” (Uddin et al., 2007). The MENT (see Figure 1) integrates internally oriented MNS processes with higher-level subjective inferences (insights) about others in order to prepare and execute appropriate social interactions (Sandrone, 2013). The MENT plays a key role in thinking and learning across the span of learners’ lifetimes; from its emergence in 2-day-old newborns (Gao et al., 2009) to its disappearance in brain-dead patients (Boly et al., 2009). While recent studies on the MNS emphasize behavioral responses to social stimulus, the MENT is thought to integrate those lower-order signals with more complex meta-processes (e.g., “reflection-in-action”), which encourage the conscious use of learning strategies related to planning, monitoring, and reflection. Reflective MENT-processes seek to reveal insights into others so that inferences may be made about the other’s inner cognitive and affective/motivational states (Shea et al., 2014).

A social-interaction becomes a *learning*-interaction when the interactants are cognitively and motivationally engaged in peer-learning activities (see Pintrich and Zusho, 2002). During a specifically learning-interaction learners formulate and consciously adapt their social strategies so they maintain intersubjectivity and sustain cooperation (Kirsh and Maglio, 1995; Black and Wiliam, 1998, 2009). It may be seen as a social game, during which the rules become known implicitly through observation and inference, and explicitly through dialog. When learners employ personal and social strategies that influence others they experience rewarding “MENT sensations” related to making progress in the social game (Schilbach et al., 2013). The causal link between peer-learning and positive emotional/motivational states means that interactive styles of learning (Black and Wiliam, 2006) are something to look forward to for students. This is particularly useful in compulsory settings where many students dislike learning (Gilman and Anderman, 2006).

The integration of signals by the MNS and MENT working together in concert is thought to be basis for the internalization of external feedback (Sandrone, 2013). Feedback signals are combined with information from memory in order to select an appropriate and timely response (Molnar-Szakacs and Uddin, 2013). This places beneficial cognitive demands on learners by requiring them to synthesize moment-to-moment feedback with prior knowledge. Synthesis requires employing the important meta-cognitive process of reflection in order to create new “schematic knowledge” (originated by Bartlett, 1932). Higher-level intersubjectivity (cognitive empathy) requires reflection on the actions and emotional states of others; including perspective taking and Theory of Mind (ToM; de Waal, 2008). The ToM regions of the human brain are synonymous with the MENT in studies on the neural basis for social interaction. Both terms refer to the substrates recruited when a learner is trying to model the insights of others during an interaction. Advanced meta-cognitive skills, such as reflection, require the recruitment of the higher MENT functions, which, with regular exposure to high-quality cooperative interactions, develop in their neuro-plasticity (Allen and Williams, 2011).

This trajectory toward higher-order social cognition is described by Decety and Jackson (2004) as a process of increasing “cognitive flexibility.” The meta-process of reflection, which develops flexibility and is itself elevated by that greater flexibility, is emphasized as pivotal to student achievement (Schön, 1987; Butler and Winne, 1995; Bose and Rengel, 2009; Clark, 2012) and a “key ingredient in the commitment to lifelong learning” (Kuiper and Pesut, 2004). Findings from the educational literature support the proposition that when peer-interaction is emphasized as an instructional strategy, the subsequent activation of the MENT plays a key role in the enhancing student engagement and the acquisition of adaptive thinking and learning strategies (Slavin, 1996; Topping, 1996; Ladyshewsky, 2001; van der Meer, 2011). This indicates that developments in the actual capacity for effective spontaneous thinking or “reflection-in-action” (Schön, 1987) depend on the frequent use of instructional strategies, which expand opportunities for student participation (Clark, 2012). When participation is underpinned by spontaneous (Guionnet et al., 2012) online peer-interaction the experience of learning together stimulates “intrinsically” rewarding MENT activity (Redcay et al., 2010; Sakaiya et al., 2013) which reinforces feelings of anticipation for learning (Salamone and Correa, 2012) and a desire to improve mastery over the rewarding meta-cognitive skills (e.g., reflection) that support achievement.

## SOCIAL INTERACTION AND THE REWARD-RELATED NETWORKS OF THE BRAIN

Reward related signals play a key role in the establishment and maintenance of social relations (Schilbach et al., 2013). Central to social relations is the notion of reciprocity (Melis and Semmann, 2010), which is also intimately connected with high social cognition (Brosnan et al., 2010). Evidence from neuroimaging and psycho-physiological studies has demonstrated “profound differences in neural processing related to the reciprocity of social interaction” (Schilbach et al., 2013). Put another way, cooperative peer-interactions stimulate directly the significant changes in brain chemistry, which influence the quality and duration of the peer-learning activity (Yamasue et al., 2009). This is proposed as a bi-directional relationship so that the positive feelings experienced by learners as they begin an interaction deepens their involvement in social- and peer-interaction which they experience as a rewarding sense of self- and social-awareness with lifelong effects.

Recent research on the production of dopamine by the mesolimbic reward system has exploded the myth that dopamine regulates positive-feelings only *when* we obtain something that satisfies us. Salamone and Correa (2012) found that dopamine neurotransmitters “fire” *before* we perform an action. The simple expectation of a cooperative interaction, based on past experience, recruits the social reward-networks in the brain. This response makes it much more likely that learners will employ social strategies which sustain the next interaction to a mutually agreeable conclusion. In a carefully structured peer-learning ecology (Clark, 2014), the automatic release of dopamine has a clear potential to reinforce peer-engagement and diminish negative psychological states, caused, for example, by negative public comparison in the classroom. However, Salamone and Correa (2012) emphasize that beneficial outcomes are not inevitable or equally distributed among the participants. The attainment of reciprocal intersubjectivity is facilitated or frustrated by the cultural practices, personal experiences, and current knowledge available to the participants. Public schools should and can organize in ways that reduce these social, cultural (e.g., linguistic) or economic inequities and support marginalized students so they remain persistent (Organization for Economic Cooperation and Development/Centre for Educational Research and Innovation [OECD/CERI], 2011; Putney and Broughton, 2011). When learners (of any age) experience social inequity or psychological threats to their self-esteem (or observe this happening to others), they divert resources away from active participation in the learning process and expend resources on efforts to avoid interaction and withdraw from the situation (Boekaerts and Corno, 2005; Black and Wiliam, 2009).

If students are to actively participate in their own learning progression it is important to help students to acquire positive volitional strategies and reinforce them by providing positive learning experiences (Black and Wiliam, 2009). Classrooms emphasize “respect, responsibility, cooperation, and caring” (Putney and Broughton, 2011) as the guidelines for community conduct; such reciprocal relationships play a key part in any community where the members expect to meet each other regularly (Henrich et al., 2003). In such communities, humans are “conditional cooperators” who discriminately prefer to learn with other cooperators but not with non-cooperators (Sakaiya et al., 2013). It is this preference, described in the next section of this article, which resides at the heart of Bandura’s (2001) “agentic” relationship. For peer-learning to flourish, the wider learning environment must be organized in ways which bring about the emotional engagement and interpersonal reciprocity required for social relationships to become learning relationships (Lave and Wenger, 1991; Putney and Broughton, 2011; Clark, 2014).

## AGENCY, RECIPROCITY, AND REWARD

“To be an agent is to intentionally make things happen by one’s actions […]. The core feature of agency enables people to play a part in their self development, adaptation, and self-renewal with changing times” (Bandura, 2001). Therefore, in a classroom setting agency refers to individual leadership during peer-interaction. When learners are engaged as active participants in cooperative peer-learning tasks they are engaged in a series of neurally rewarding “agentic” interactions. The precise terms of an agentic relationship are negotiated between the participants. This negotiation (or reciprocation) continues for the duration of an interaction, and regulates equality and mutuality (Storch, 2002) among student-peers. When discursive control is equitably circulated every member of the learning community or group has contributed equally and mutually their ideas and opinions are perceived as equally valuable and they should, at least in theory, have experienced equal reward.

However, the search for equity is not the concern of young learners during an interaction. Social neuroscience has discovered that people are very sensitive to the reward they experience when they have influence over an interaction (Fiske and Dépret, 1996; Schilbach et al., 2013). In practice this means that each learner, to some extent, prefers to play the role of influencer or controller because dopamine is released when they are afforded the opportunity to lead the interaction. This requires the cooperation of the other “players” in the social game which is why many social interactions are unrewarding and unpleasant experiences when cooperation is withheld. In social situations where they feel that they are guiding the other they feel valued and the reward-systems of the brain induce a sense of well-being and self-esteem. Accordingly, in classroom studies conducted by Hedin (1987) it was observed that “the experience of being needed, valued, and respected by another person produced a new view of self as a worthwhile human being.” This is *agentic equity*; a term, which refers to, equally distributed leadership among the interactants. The actual extent of equity between the interactants depends upon interactive turn-taking and the circulation of reciprocal feedback among children. Ideally, everybody will have experienced a sense of self-worth associated with being valued at some point during the interaction.

Learning relationships of this quality do not occur spontaneously. They cannot be attained without: (a) persistent modeling by teachers; (b) frequent opportunities to participate in structured or “scaffolded” peer-learning; or, (c) if required, more prescriptive interventions and training programs which support the socio-emotional needs of learners (Blatchford et al., 2006; Collaborative for Academic, Social, Emotional Learning [CASEL], 2006). They take turns in a way known as the “interactive turn” by directing each other in accordance with Storch’s (2002) notion of equality as a necessary aspect of the privileged learning “contract” between them. The ventral striatum (VS; see Figure 1) in the midbrain (MENT regions) and other reward processing structures are furthermore recruited during cooperative (Schilbach et al., 2013) and spontaneous (Guionnet et al., 2012) interaction.

## THE DOPAMINERGIC PATHWAY IN SOCIAL REWARD

Recent evidences have emerged that cooperative peer-interaction recruits the mesolimbic dopamine reward system in the human brain, providing a feeling of fulfillment to the learners engaged in the interaction (Redcay et al., 2010; Krill and Platek, 2012; Sakaiya et al., 2013; Pfeiffer et al., 2014). The VS is particularly important in the dopaminergic pathway (see Figure 1). It receives rich dopaminergic input from the midbrain (Tabibnia and Lieberman, 2007) and integrates actions with reward, thus capable of translating social information into coding of new behavior, including learning (Báez-Mendoza and Schultz, 2013). Cooperative and shared social context seems to particularly activate the VS (Tabibnia and Lieberman, 2007; Fareri et al., 2012; Guionnet et al., 2012); Schilbach et al. (2010) even mention a “VS effect” that was triangulated by questionnaire responses and which confirmed that cooperative social interaction “was experienced as more pleasant and less effortful” than doing the opposite of their partner. The amygdala also plays a key role in reward-processing activity (Baxter and Murray, 2002) and use of reward to motivate the use of social learning strategies (Murray, 2007).

Developments in functional magnetic resonance imagining (fMRI) elucidate on the connection between reciprocal peer-interaction and the amygdala, which coordinates with other reward related structures; including the ventral tegmental area (VTA). For example, Sakaiya et al. (2013) found a link between amygdala activity and the cooperative learning interactions, which regulate agentic equity. Amygdala activation was greater for cooperative social strategies than for interactions, which the interactants perceived as unpredictable. When the participants in Sakaiya et al. (2013) study were exposed to unstable interaction strategies, when reciprocation and non-reciprocation could not be predicted, they reported a corresponding loss of insight into their partner’s intentions. The consequence of this lack of mutual empathy was a diminished desire to continue the learning interaction. Consequently, learning ceases along with the motivation to do so. This was confirmed by interviews in which the participants in Sakaiya et al.’s (2013) study expressed a preference for changing partners or discontinuing the interaction entirely.

Reciprocity, mutuality, and shared intention are key components for an efficient co-regulation of spontaneous social interaction (Nadel and Dumas, 2014). Cooperative interactions are especially of this kind and rewarding experiences usually emerge between two or more intentionally supportive peers (cf. Vygotsky, 1978, 1987). When interactants use cooperative social strategies, brain structures associated with the dopaminergic reward system are indeed more active (Redcay et al., 2010; Krill and Platek, 2012; Sakaiya et al., 2013; Schilbach et al., 2013); The same effect is observed for the anticipation of an interaction (Salamone and Correa, 2012), when interactants develop a common understanding in regard to motive and the goals and the pay off so that all participants achieve the same goal successfully (Rilling et al., 2002; Decety et al., 2004). In all those cases, dopamine thus creates and sustains the positive emotional states during social interaction and, in the case of learning, contributes to the intrinsic motivation of learners.

## THE DEVELOPMENTAL TRAJECTORY OF THE MENT AND PEER-LEARNING

Interactive peer-dialog, characteristic of learners who are able to employ “reflection-in-action,” is founded upon the level of “spontaneous activity” (Fair et al., 2008) taking place across the MENT regions of the human brain. Fair et al. (2008) posit that more mature MENT substrates exhibit a higher level of spontaneity in response to social affordances. Some studies suggest that the MENT’s maturity is connected to the chronological age of the learner. For example, Bahrami et al. (2010) emphasize the finding that enhancements in the reflective and introspective aspects of social cognition accrue throughout learners’ lives.

When investigating the earliest years of cognitive development, Fransson et al. (2008) could not detect a complete MENT system in the infants in their study. This finding was elaborated on by the pioneering work of Fair et al. (2008) who found the MENT to be only “sparsely functionally connected” at 7–9 years of age. The same study emphasized a growth-trajectory of the MENT, suggesting, “over development, these regions integrate into a cohesive, interconnected network.” In a later study, Washington et al. (2014) also investigated the structural connectivity between the MENT sub-structures. Like Fair et al. (2008), they found weak structural connectivity in 7–9 year olds, so that these regions of the brain still did coordinate efficiently. Further, Washington et al. (2014) discovered that the MENT regions of the brain do not begin to work together in concert (as they do in adults) until ages 11–13. Despite these structural weaknesses across the MENT regions in pre-teens and children of elementary school age, learners as young as 8 years can routinely recall and restructure (i.e., reflection) the content of past and present thinking and learning (Fair et al., 2008).

The foundational work of Fair et al. (2008) supports the conclusion that the MENT is a relatively functional structure at the age when meta-cognitive skills (i.e., reflection) become developed. Supekar et al. (2010) conducted a similar study to confirm the earlier study conducted by Fair et al. (2008). Supekar et al. (2010) found that by the age of 8, some connections between MENT substrates were mature and others were in an earlier state of maturation (cf. Vygotsky, 1978, 1987). In accordance with the earlier study by Fair et al. (2008), Supekar et al. (2010) found that despite sparse functional connectivity, 8-year-old children can reach “adult-like levels” in sub-structures implicated in social cognition. Those results follow a more general trend of brain networks that mature from a “local” organization to a “distributed” organization (Fair et al., 2009). Overall, these emerging findings indicate that children should be meaningfully assessed for their ability to establish agentic equity at around the age of 8 years old; an age at which peer-learning may begin to become an effective instructional strategy. On this latter point, the early activation of the dopaminergic reward linked with social interaction encourages peer-learning partnerships, which Reyes and Elias (2011) found to establish “the most powerful integration of protective processes”; processes, which protect young learners from academic disaffection and failure later in life.

## CONCLUSION

Johnson and Johnson (1983) emphasize that,

“[…] there is no type of task on which cooperative efforts are less effective than are competitive or individualistic efforts, on most tasks[…]concept attainment, verbal problem-solving, categorization, spatial problem-solving, retention and memory, motor, guessing-judging-predicting, cooperative efforts are more effective in promoting achievement.”

Scientific evidence indicates that effective peer interactions are characterized as stable, equitable, mutually engaging and reciprocal interactions. Cooperative social interaction depends upon the establishment and maintenance of intersubjectivity; a persistent process that sustains any learning-interaction for its entire duration. When learners work together cooperatively they rely on the moment-to-moment integration of lower-order motor cues in the MNS and higher-order reflection and projection processes in the MENT regions of the human brain. Redcay et al. (2010) found that those key neural structures recruited for everyday social interaction (right temporo-parietal junction, anterior cingulate cortex, and right superior temporal sulcus) are “consistently linked” to the activation of the dopaminergic reward system (amygdala and VS; see Figure 1). The same study emphasized “the powerful and pervasive drive” for humans to seek out social interactions, and reiterated that contingent interactions with another person recruits the reward systems (Guionnet et al., 2012; Krill and Platek, 2012; Sakaiya et al., 2013; Schilbach et al., 2013).

Cooperative peer-learning can be described as the instructional use of pairs or small groups so that students work together to maximize their own and each other’s learning. It may be contrasted with competitive contexts and individualistic contexts (Johnson, 2009). Significantly, socio-constructivist contexts which promote an interactive style of knowledge construction sustain and deepen participation (cf. Lave and Wenger, 1991) between learners, and provide dopaminergic reward in anticipation of (Salamone and Correa, 2012) and during the learning-interaction (Redcay et al., 2010; Guionnet et al., 2012; Krill and Platek, 2012; Sakaiya et al., 2013; Schilbach et al., 2013), which cease after the interaction has ended. These emerging findings from within social neuroscience inform our understanding of the value of peer-learning in classrooms across multiple contexts. The opportunities for future research into the “dark matter” of social neuroscience are potentially vast in their scope and implication. A current finding of general relevance is that the brain responds differently depending on whether learning entails passive observation or active participation (Schilbach, 2014). Both learning “stances” involve intricate processes of internalization, reflection, and social knowledge construction, which together promote the intrinsic motivation required for successful outcomes in public schools.

### Conflict of Interest Statement

The authors declare that the research was conducted in the absence of any commercial or financial relationships that could be construed as a potential conflict of interest.
